# Object Detection Based on Swin Deformable Transformer-BiPAFPN-YOLOX

**DOI:** 10.1155/2023/4228610

**Published:** 2023-03-09

**Authors:** Peicheng Shi, Xinhe Chen, Heng Qi, Chenghui Zhang, Zhiqiang Liu

**Affiliations:** School of Mechanical Engineering, Anhui Polytechnic University, Wuhu 241000, China

## Abstract

Object detection technology plays a crucial role in people's everyday lives, as well as enterprise production and modern national defense. Most current object detection networks, such as YOLOX, employ convolutional neural networks instead of a Transformer as a backbone. However, these techniques lack a global understanding of the images and may lose meaningful information, such as the precise location of the most active feature detector. Recently, a Transformer with larger receptive fields showed superior performance to corresponding convolutional neural networks in computer vision tasks. The Transformer splits the image into patches and subsequently feeds them to the Transformer in a sequence structure similar to word embeddings. This makes it capable of global modeling of entire images and implies global understanding of images. However, simply using a Transformer with a larger receptive field raises several concerns. For example, self-attention in the Swin Transformer backbone will limit its ability to model long range relations, resulting in poor feature extraction results and low convergence speed during training. To address the above problems, first, we propose an important region-based Reconstructed Deformable Self-Attention that shifts attention to important regions for efficient global modeling. Second, based on the Reconstructed Deformable Self-Attention, we propose the Swin Deformable Transformer backbone, which improves the feature extraction ability and convergence speed. Finally, based on the Swin Deformable Transformer backbone, we propose a novel object detection network, namely, Swin Deformable Transformer-BiPAFPN-YOLOX. experimental results on the COCO dataset show that the training period is reduced by 55.4%, average precision is increased by 2.4%, average precision of small objects is increased by 3.7%, and inference speed is increased by 35%.

## 1. Introduction

Object detection represents one of the major concepts in the field of computer vision. In everyday life, advanced object detection technology can be used for intelligent vehicle environment perception tasks to facilitate travel; in enterprises, it can be used for normal operations in specific scenarios, such as parks and ports; in modern national defense, it contributes to better completing offensive and defensive tasks. You Only Look Once (YOLO) is a representative algorithm in object detection, employing a convolution neural network (CNN) as the backbone for feature extraction. For example, YOLOv3 [1] and YOLOX [2] use Darknet-53 [1] as their backbone, while YOLOv4 [3] and YOLOv5 [4] use CSPDarknet53 [5]. However, these techniques are translation-invariant, locality-sensitive, and lacking a global understanding of images. Furthermore, CNN-based models use the pooling layers strategy for a dimensionality reduction to reduce the computational cost, which causes loss of a significant amount of meaningful information, such as the precise location of the most active feature detector.

The Transformer is a model that relies entirely on the self-attention for natural language processing. Recently, a Transformer [6] achieved superior performance in language modeling and machine translation tasks. The Transformer-based model has a larger receptive field than that of the CNN, globally models the entire image and has a global understanding of images, which prompts people to start exploring the application of the language Transformer to the visual field. Several studies [7–12] modeled the vision task as a dictionary lookup problem with learnable queries and used the Transformer encoder as a task-specific head on top of the CNN backbone. In image classification tasks, one study [13] was the first to propose a Vision Transformer (ViT) method, by directly employing a standard Transformer to images by transforming images into patches instead of focusing on pixels, then inputting them to the Transformer encoder. As described in research [14], the image patches are treated as tokens in natural language processing applications. This leads to highly competitive results on the ImageNet dataset.

In the existing visual transformer research, ViT [13] is suitable for image classification tasks, which is an interesting and meaningful attempt to replace the CNN backbone with a convolution-free model. Although ViT [13] is applicable to image classification, its direct adaptation to pixel-level dense predictions such as object detection is challenging, because (1) its output feature map is single-scale and low-resolution, and (2) the excessive number of keys to attend per query patch yields high-computational cost and also increases the risk of overfitting.

To avoid excessive attention computation, existing studies [15–19] leveraged carefully designed efficient attention patterns to reduce the computational complexity. Studies [17, 20] downsample the key and value feature maps to save on computation costs. Although this method is effective, it is likely that relevant keys and values are dropped, while less important ones are kept, which causes important features to be lost. The attention mechanism in one study [21] adaptively localized the object regions; however, its random reference points weakened the correlation between features, resulting in feature loss. The Swin Transformer [22, 23] adopts shift window-based attention to restrict attention in local windows. Despite their effectiveness, the hand-crafted attention patterns are data-agnostic, which limits their self-attention modeling ability, reduces feature extraction ability, and is suboptimal. Moreover, self-attention requires a long training period for adaptive learning to focus on object regions on the images, which results in slow convergence.

Notably, deformable convolution [24, 25] can learn deformable receptive fields and attend to flexible spatial locations conditioned on input data. This was shown to be effective in selectively attending to more informative regions on a data-dependent basis. However, its deformable offsets make the computational cost quadratic with the image size. Inspired by deformable convolution [24, 25], Deformable DETR [8] improves the convergence and decreases the computation complexity of DETR [7] by selecting a small number of keys for each query. However, its attention is not suited to a visual backbone for feature extraction, because the lack of keys restricts representational power.

To solve the above problems, we pursue the design of a novel attention mechanism that adaptively focuses on important object regions and ignores nonimportant features, to improve the modeling ability and speed of the attention mechanism. With this attention mechanism, our Transformer has powerful feature extraction capabilities for important object regions and fast convergence capabilities during training for object detection tasks. Along with the above ideas, we propose the Reconstructed Deformable Self-Attention based on important regions, which shifts attention to important regions to capture more informative features for global modeling. Specifically, first, we use the grid points composed of patches as patch grid points; then, we use the query as input of the offset network to generate the offsets corresponding to all patch grid points; finally, the patch grid points combine the offsets and transfer key/value to the important regions. In this manner, the Reconstructed Deformable Self-Attention, which depends on the data pattern, can focus on more important and relevant object regions to capture a larger number of features more efficiently. This improves the modeling ability and effectively shortens the modeling time. Based on the Reconstructed Deformable Self-Attention, we propose the Swin Deformable Transformer backbone. Compared with the Swin Transformer, the Swin Deformable Transformer can retain more important keys and values and ignore irrelevant ones, resulting in higher flexibility and efficiency. This significantly reduces the training time while maintaining efficient feature extraction capabilities.

The role of neck in YOLOX is to integrate features of different stages and scales, such that YOLOX has the ability to represent multiscale features. YOLOX's neck is PAFPN [26], which fuses different input features by summation. Different input features have different resolutions, and their contribution to the output features after fusion is not uniform. In actual calculation, the weights of each input feature are the same, which lessen the contribution of important features. To address the above issue, we use BiPAFPN [27] as the neck for YOLOX. BiPAFPN learns the importance of different input features by introducing learnable weights, thereby enhancing the contribution of important features.

In summary, our contributions are as follows:

(1) We propose a novel Reconstructed Deformable Self-Attention based on important regions, which shifts attention to important object regions in the image, ignores unimportant features, and then performs efficient visual modeling of target features. (2) Based on the Reconstructed Deformable Self-Attention, we propose a novel backbone Swin Deformable Transformer, which speeds up the convergence while improving the feature extraction ability. (3) We design a novel transformer-based object detection network: Swin Deformable Transformer-BiPAFPN-YOLOX, which uses the Swin Deformable Transformer as the backbone of YOLOX and BiPAFPN as the neck of YOLOX. This study proposes the transformer as the backbone of YOLO for the first time and obtains superior performance to the CNN.

The unified framework of CV and NLP was shown to promote the common development of the two fields. The leap from the language transformer to the visual transformer promotes the joint modeling of visual and textual information. The extensive applications of the transformer in various fields imply its realization of the functions of multimodal colearning and unified modeling in the future.

## 2. Related Work

### 2.1. CNN

CNN is a standard network in the field of computer vision. Since the introduction of AlexNet [28], CNN has become a mainstream. A previous study [29] proposed a CNN-based detection network used for behavior analysis and violence detection in the industrial Internet of Things. Another study [30] proposed a 101-layer backbone ResNeXt-101 for feature extraction. Yet another study [31] proposed a lightweight backbone MobileNet for feature extraction, while reference [32] proposed a depthwise separable convolution [33] based network, which produces real-time high quality density maps and effectively counts people in extremely overcrowded scenes. However, these CNN-based networks lose meaningful information when employing the dimensionality reduction mechanism. Furthermore, this method requires powerful GPUs and a large amount of data for effective training. Recent studies found that the Transformer-based network has a larger receptive field and modeling ability than CNN and can solve the above problems of CNN.

### 2.2. Transformer

The pioneering work of visual transformer ViT [13] firstly proposes to apply Transformer architecture on nonoverlapping image patches and achieves an impressive speed-accuracy trade-off on image classification compared to CNN. Meanwhile, ViT [13] requires large-scale training datasets (i.e., JFT-300M) and a large number of training epochs to perform efficiently. DeiT [34] introduces several training strategies that allow ViT to be effective using the smaller ImageNet-1K [35] dataset. The results of ViT on image classification are encouraging; however, its architecture is not suitable for use as a general-purpose backbone network on dense vision tasks or when the input image resolution is high, due to the attention low-resolution feature maps and the quadratic increase in complexity with image size.

After the introduction of ViT [13], improvements focused on learning multiscale features for dense prediction tasks. CvT [36] adopts convolution in the tokenization process and utilizes stride convolution to reduce the computational complexity of self-attention. SepViT [37] uses novel window token embedding and grouped self-attention to model the attention relationship among windows with negligible computational cost. PVTv2 [17] reduces the computational complexity of PVTv1 [20] to linear by adding linear complexity attention layers, overlapping patch embedding, and convolutional feed-forward network.

Subsequently, focus has shifted to efficient attention mechanisms for multiple computer vision downstream tasks. These attention mechanisms include global tokens [15, 38, 39], focal attention [18], and dynamic token sizes [40]. Reference [41] sequentially proposes the spatial separable self-attention and cross-shaped window self-attention based on the hierarchical architecture. Deformable convolution [24, 25] is a powerful mechanism to attend to sparse spatial locations conditioned on input data. Inspired by deformable convolution, Deformable DETR [8] combines the advantages of the sparse spatial sampling of deformable convolution and the relation modeling capability of transformer, effectively shortening the training period. However, its attention is not suited to a visual backbone for feature extraction, as the attention in Deformable DETR comes from simply learned linear projections, and keys are not shared among query tokens. Cswin transformer [16] and Swin Transformer [22, 23] adopt the windowed attention and show improvements on downstream tasks. However, these attentions limit the ability to model long range relations, resulting in poor feature extraction results and slow convergence speed. Although attention and attention-based Transformer backbone are constantly being improved, they nevertheless fail to fully improve the ability of the attention to model target features, as well as to improve the feature extraction ability and convergence speed of the Transformer backbone. Based on this research, we propose our network to overcome the outstanding challenges.

### 2.3. Neck

Multiscale feature aggregation represents features of different resolutions more effectively. PANet [26] adds a bottom-up path aggregation network on the top of FPN [42]. This solves the problem of restricted information flow in FPN [42]; however, important features in PANet [26] input are weakened. NAS-FPN [43] leverages neural architecture search to automatically design feature network topology. Despite achieving better performance, NAS-FPN [43] requires thousands of GPU hours during the search. BiPAFPN [27] introduces learnable weights to learn the importance of different input features, while repeatedly applying top-down and bottom-up multiscale feature fusion. It strengthens the contribution of important features and performs feature fusion more efficiently.

### 2.4. Head

The detection head is responsible for predicting bounding boxes and object classes. The two-stage detector proposed in reference [44] holds high precision but low real-time performance. The one-stage detector proposed in reference [45] has high real-time performance but low precision and performs poorly on small-scale objects. YOLOX [2] proposed an object detection network based on the anchor-free, decoupled head, and label assignment strategy SimOTA, which avoids over-reliance on techniques such as anchor clustering [46] and grid sensitivity [47] significantly simplifying the detector and gaining its advanced performance.

## 3. Method

### 3.1. Overall Architecture

**Figure 1:**
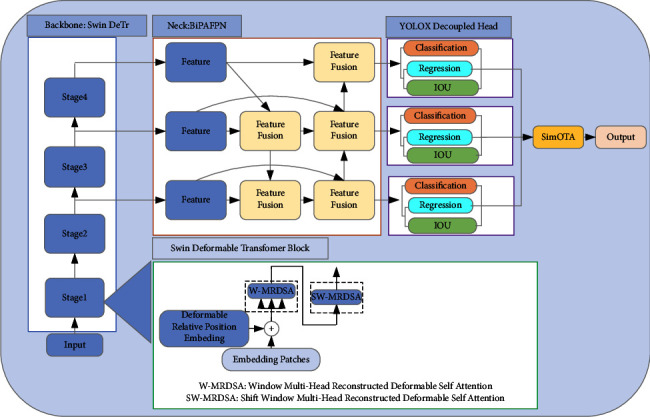
Swin Deformable Transformer-BiPAFPN-YOLOX network architecture. Swin DeTr denotes Swin Deformable Transformer.

We propose Reconstructed Deformable Self-Attention as the attention module of the Swin Deformable Transformer and based on the Reconstructed Deformable Self-Attention, we propose the Swin Deformable Transformer. We use the Swin Deformable Transformer as the backbone, BiPAFPN as the neck, and YOLOX as the detection head. The entire object detection network architecture is shown in Figure 1. Firstly, the images are passed to the four-stage backbone Swin Deformable Transformer to complete multiscale feature extraction; then, the features of different scales are transmitted to the neck BiPAFPN for multiscale feature aggregation to achieve a comprehensive understanding of images; finally, the features are transmitted to the YOLOX detection head, to predict the class, location, and bounding box confidence of objects.

### 3.2. Reconstructed Deformable Self-Attention

**Figure 2:**
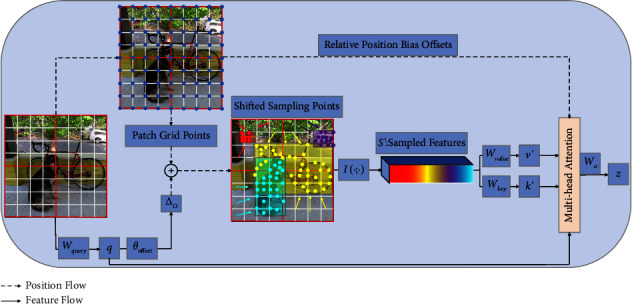
Reconstructed Deformable Self-Attention. Red boxes depict windows, gray boxes are patches, and blue dots are patch grid points. Red, purple, yellow, and green regions represent regions of importance, showing that the patch grid points move to important regions.

The Reconstructed Deformable Self-Attention based on important regions in the Swin Deformable Transformer is shown in Figure 2. It models the relations between patches under the guidance of important regions in the feature map. These important regions are determined by shifted sampling points. Due to the existence of shifted sampling points, these regions are assigned more local intensive attentions than other regions, which improve the modeling ability and capture important features more accurately.

First, features are input to generate patch grid points.

#### 3.2.1. Patch Grid Points

Patch grid points refer to the four grid points on each patch, and the important features in each patch are located through the bounding box composed of four points to ensure that they are not lost. On the one hand, this prevents the information loss of the entire feature map and reduces the computational cost and time cost of remeshing. On the other hand, because patches are located within the shifted windows, the shifted windows can interact with each other, which enhance the feature-to-feature correlation. The values of patch grid points are linearly spaced 2D coordinates {(0,0), ⋯, (*P*_*H*_ − 1, *P*_*W*_ − 1)}, *P*_*H*_, and *P*_*W*_ are the number of patches in the height and width directions, respectively. We normalize them to the range [−1, +1] according to the grid shape *P*_*H*_ × *P*_*W*_, where (−1, −1) pinpoints the top-left corner and (+1, +1) the bottom-right corner.

Second, to obtain the offsets of patch grid points, we project the feature through the projection matrix *W*_query_ to obtain query (*q*) as in equation (1), and then input *q* to the offset net *θ*_offset_() to generate an offset ∆*o* as in equation (2). Under the guidance of important regions in the feature map, patch grid points combined with offsets become shifted sampling points and migrate to important regions.

Third, feature maps are sampled at the position of shifted sampling points to obtain the sample features *S*′ as in equation (3). Then, *S*′ are projected by the projection matrices *W*_key_ and *W*_value_ to obtain shifted keys and shifted values respectively, as in equation (4).

(1) $$\begin{array}{c} q=xW_{query}\text{,} \end{array}$$

(2) $$\begin{array}{c} ∆o=s\;\tan\;h\left[\theta_{offset}\left(q\right)\right]\text{,} \end{array}$$

(3) $$\begin{array}{c} S^{\prime }=I\left(x;p+∆p\right)\text{,} \end{array}$$

(4) $$\begin{array}{c} k\prime =S\prime W_{key}\text{,} \end{array}$$

where *W*_query_, *W*_key_, and*W*_value_ are the projection matrices, *k*′and*v*′ are the embeddings of shifted keys and shifted values, respectively.

For equation (3), specifically, we set the sampling function *I*(∙; ∙) to a bilinear interpolation to render it differentiable as in equation (5):

(5) $$\begin{array}{c} I\left(z;\left(p_{x},p_{y}\right)\right)=\underset{\left(r_{x},r_{y}\right)}{\sum }g\left(p_{x},r_{x}\right)g\left(p_{y},r_{y}\right)z\left[r_{y},r_{x},\text{:}\right]\text{,} \end{array}$$

where *g*(*p*_*x*_, *r*_*x*_) = max   (0,1 − |*p*_*x*_ − *r*_*x*_|) and (*r*_*x*_, *r*_*y*_) indexes all the locations on the feature map *z* ∈ *ℝ*^*H*×*W*×*C*^. (*p*_*x*_, *p*_*y*_) are the coordinates of the patch grid points.

Fourth, we take relative position offsets *R* and compute the attention of *q*, *k*, and *v*. The output of single-head attention is shown in equation (6).

(6) $$\begin{array}{c} z^{\left(m\right)}=softmax\left(\frac{q^{\left(m\right)}k^{{\prime \left(m\right)}^{⊺}}}{\sqrt{d}}+I\left(\hat{B};R\right)\right){v^{\prime }}^{\left(m\right)}\text{,}\\ m=1,\ldots ,M\text{,} \end{array}$$

where *m* represents the attention head index, *d* is the dimension of each attention head, $\hat{B}\in {\mathbb{R}}^{\left(2H-1\right)\times \left(2W-1\right)}$ is the relative position bias table [22], from which we can index the relative position bias *B*; $I\left(\hat{B};R\right)\in {\mathbb{R}}^{HW\times P_{H}P_{W}}$ is the interpolation of $\hat{B}$. We concatenate all attention heads and use *W*^*o*^ to project them to obtain multihead attention as in equation (7).

(7) $$\begin{array}{c} z=Concat\left(z^{\left(1\right)},z^{\left(M\right)}\right)W^{o}\text{.} \end{array}$$

#### 3.2.2. Point Box Offset

**Figure 3:**
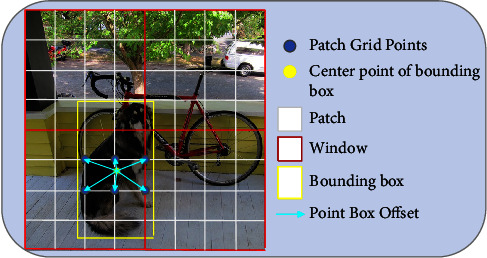
Point box offset.

Since the Reconstructed Deformable Self-Attention extracts image features around patch grid points, we use the detection head to predict point box offsets between the center of the bounding box and patch grid points, as shown in Figure 3. The implementation is as follows: we take the patch grid points as the initial inference points for the center point of the bounding box. Then, we let the detection head predict the offsets of the patch grid points relative to the actual center point of the bounding box, as shown in equation (8).

(8) $$\begin{array}{c} \left.\begin{array}{c} p=\left(p_{x},p_{y}\right)\text{,}\\ \hat{b}=\left\{\sigma \left(b_{x}+\sigma^{-1}\left(p_{x}\right)\right),\sigma \left(b_{y}+\sigma^{-1}\left(p_{y}\right)\right),\sigma \left(b_{w}\right),\sigma \left(b_{h}\right)\right\} \end{array}\right\}\text{,} \end{array}$$

where *p* is the patch grid point, $\hat{b}$ is the offset, *b*_{*x*, *y*, *w*, *h*}_ ∈ *ℝ* is predicted by the detection head. *σ* and*σ*^−1^ denote the sigmoid and the inverse sigmoid function, respectively. The usage of *σ* and*σ*^−1^ is to ensure $\hat{b}$ is of normalized coordinates, as $\hat{b}\in \left[0,1\right]$. In this way, the learned Reconstructed Deformable Self-Attention has strong correlation with the predicted bounding box, which improves the detection precision.

### 3.3. Backbone: Swin Deformable Transformer

**Figure 4:**
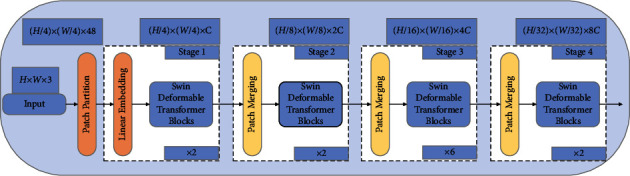
Swin Deformable Transformer network architecture.

The backbone Swin Deformable Transformer based on the Reconstructed Deformable Self-Attention contains four stages, and its architecture is shown in Figure 4.

The patch partition module splits the input image of size *H* × *W* × 3 into uniformly distributed patches to be received by the Transformer. After patch partition, the patches of size (*H*/4) × (*W*/4) × 48 are obtained. After entering stage1, the linear embedding module projects the patches to any dimension of input of the Swin Deformable Transformer Blocks, where (*H*/4) × (*W*/4) × *C*. Finally, the feature is passed into the Swin Deformable Transformer Blocks of stage 1.

#### 3.3.1. Swin Deformable Transformer Blocks

**Figure 5:**
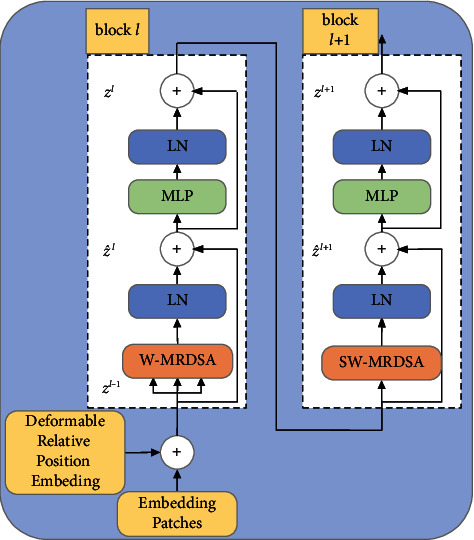
Swin Deformable Transformer Blocks.

The structure of the Swin Deformable Transformer Blocks is shown in Figure 5. Block *l* performs the Window Multihead Reconstructed Deformable Self-Attention (W-MRDSA), after which, block *l* *+* 1 performs the Shift Window Multihead Reconstructed Deformable Self-Attention (SW-MRDSA), with the two blocks as a computation unit.

First, the output embedding patches of linear embedding in Figure 4 and position encode are passed to W-MRDSA. We use deformable relative position embedding as the position encode. On the one hand, it can cover all offsets, and on the other hand, it is responsible for encoding the specific position of the input sequence to prevent embedding patches from being out of order. Second, W-MRDSA is calculated by equation (9). The input of W-MRDSA is a series of key, query, and value vectors. We use the scaled cosine attention method to calculate the similarity between queries and keys, and obtain the weight *A*_*i*_ corresponding to the key. We use the SoftMax function to normalize the weight to obtain the weight coefficient *C*_*i*_. We perform weighted summation on the value to obtain the value of self-attention SA. We project the concatenated outputs of all self-attention to obtain the outputs of W-MRDSA.

(9) $$\begin{array}{c} \left.\begin{array}{c} A_{i}=Similarity\left({key}_{i},query\right)=\frac{\cos\;\left({key}_{i},query\right)}{\tau }+B_{ij}\\ C_{i}=Softmax\left[Similarity\left({key}_{i},query\right)\right]\\ SA=\overset{I}{\underset{i=1}{\sum }}Softmax\left[Similarity\left({key}_{i},query\right)\right]\times value\\ W-MRDSA=Concat\left({SA}_{head1},{SA}_{head2},{SA}_{headn}\right)W^{o} \end{array}\right\}\text{.} \end{array}$$

In equation (9), *B*_*ij*_ is the relative position bias between pixel *i* and pixel; *τ* is a learnable scalar with a value greater than 0.01; *W*^*o*^ being the projection matrix. The scaled cosine attention method stabilizes the training process and improves the precision. Thirdly, features are passed to the normalization layer (LN). The data must be normalized before training the neural network, which speeds up the training and improves stability of the training process. We employ the res-post-norm method: placing LN before SA avoids excessive activation amplitudes between layers, stabilizing the training process and improving precision. Fourth, the features are passed into the multilayer perceptron (MLP), such that the nonlinear model realizes linear transformation of the features dimension. The features are then passed into the next LN. There are two residual connections in each block in Figure 5, used to prevent network degradation during training, which increases the training error. Fifth, the features are transferred to block *l* *+* 1, and subsequently, the SW-MRDSA is calculated. The subsequent processes are the same as those of block *l*.

The calculation of two consecutive Swin Deformable Transformer Blocks is shown as equation (10).

(10) $$\begin{array}{c} \left.\begin{array}{c} {\hat{z}}^{l}=W-MRDSA\left(\text{LN}\left(z^{l-1}\right)\right)+z^{l-1}\\ z^{l}=MLP\left(\text{LN}\left({\hat{z}}^{l}\right)\right)+{\hat{z}}^{l}\\ {\hat{z}}^{l+1}=SW-MRDSA\left(\text{LN}\left(z^{l}\right)\right)+z^{l}\\ z^{l+1}=MLP\left(\text{LN}\left({\hat{z}}^{l+1}\right)\right)+{\hat{z}}^{l+1} \end{array}\right\}\text{,} \end{array}$$

where ${\hat{z}}^{l}$ and *z*^*l*^ are the output features of (S)W-MRDSA and MLP modules in block *l*, respectively.

The computational cost of MRDSA is calculated by equation (11).

(11) $$\begin{array}{c} \Omega \left(MRDSA\right)=2HW\left(P_{H}+1\right)\left(P_{W}+1\right)C+2HWC^{2}+2\left(P_{H}+1\right)\left(P_{W}+1\right)C^{2}+\left(k^{2}+2\right)\left(P_{H}+1\right)\left(P_{W}+1\right)C\text{,} \end{array}$$

where HW is the product of the height and width of each image, *C* is the dimension, *P*_*H*_ and*P*_*W*_ are the number of patches in the height and width directions, and *k* is the number of sampling keys. The total computational cost is linear to the image size. Thus, our proposed Swin Deformable Transformer is suitable for dense prediction vision tasks that require input high-resolution images.

**Figure 6:**
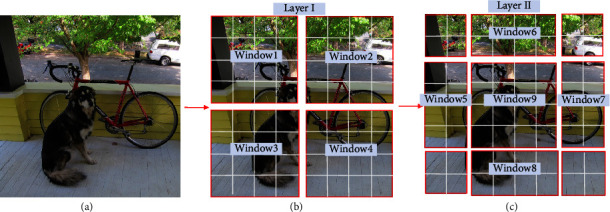
Shift windows. Gray boxes represent patches and red boxes represent windows.

The Swin Deformable Transformer divides the image into windows and calculates attention within them. Simultaneously, it establishes the connection based on shifted windows, which enables the interaction between the windows of each layer. At this time, self-attention based on shifted windows has a global modeling capability and can capture more information, as shown in Figure 6.

Layer I uses a regular window partition strategy, where the image is divided into four windows and each window has 4 × 4 patches. After the regular window partition of Layer I is cyclically shifted to the upper left, new windows of layer II are generated. Self-attention of the new windows crosses the boundary of the regularly partitioned ones, making the windows interact with each other, and thus, the global modeling ability is obtained. For example, window5 enables window1 to interact with window3, window9 enables window1, window2, window3, and window4 to interact with each other.

#### 3.3.2. Patch Merging

**Figure 7:**
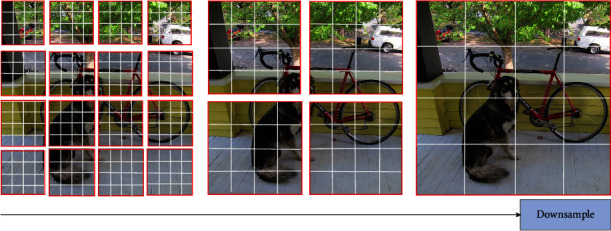
Patch merging.

In stage 2, we build the patch merging mechanism to generate multiscale features, as shown in Figure 7. First, patch merging splices four adjacent windows into a new window in the dimension of C, and the new window has a larger receptive field. The four-fold downsampling reduces the number of patches to the original 1/4, and the dimension of the patches becomes 4*C*, such that the dimension of the features after splicing is (*H*/8) × (*W*/8) × 4*C*. Then, the channels of features are reduced to 2*C* using the convolution of 1 × 1, such that the dimension of the features become (*H*/8) × (*W*/8) × 2*C*. Second, the Swin Deformable Transformer Blocks transform the features, and the size of the transformed features remains (*H*/8) × (*W*/8) × 2*C*. Similarly, the feature dimensions output by stage 3 and stage 4 are (*H*/16) × (*W*/16) × 4*C*, (*H*/32) × (*W*/32) × 8*C*, respectively, and the feature dimensions output by stage 1 and stage 2 are (*H*/4) × (*W*/4) × *C* and (*H*/8) × (*W*/8) × 2*C*, respectively. Thus, hierarchical multiscale features are formed.

### 3.4. Neck: BiPAFPN

**Figure 8:**
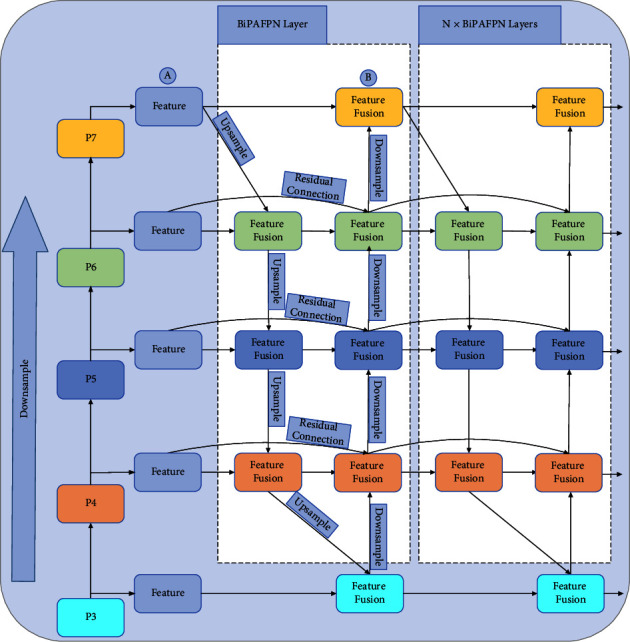
BiPAFPN network architecture.

BiPAFPN [27] is a lightweight and efficient network that enables simple and fast multiscale feature fusion. The structure of BiPAFPN is shown in Figure 8 in comparison with PAFPN [26], and its main features are as follows.

First, BiPAFPN removes nodes that only have one input edge. For example, it removes the one-way node between A and B in Figure 8. This is because if a node has only one input edge with no feature fusion, it has less contribution to the feature network that aims at fusing different features. This forms a bidirectional information transfer network of upsampling and downsampling on the PAFPN. Second, BiPAFPN adds residual connections from the original input to output node if they are at the same level, in order to fuse more features. Third, it treats each bidirectional (top-down and bottom-up) path as one feature layer and repeats the same layer multiple times to enable further high-level feature fusion.

Different input features have different resolutions, and they usually contribute to the output feature unequally. Thus, we add an additional weight for each input, and let the network learn the importance of each input feature. Finally, we adopt a fast normalized fusion strategy, as shown in equation (12):

(12) $$\begin{array}{c} O=\underset{i}{\sum }\frac{w_{i}}{\epsilon +\sum_{j}w_{j}}∙I_{i}\text{,} \end{array}$$

*w*_*i*_ is a learnable weightand *ϵ* = 0.0001 can avoid numerical instability.

After the *P*_6_ level feature fusion in Figure 8, the output is shown in equations (13) and (14):

(13) $$\begin{array}{c} P_{6}^{td}=DSConv\left(\frac{w_{1}∙P_{6}^{in}+w_{2}∙Resize\left(P_{7}^{in}\right)}{w_{1}+w_{2}+\epsilon }\right)\text{,} \end{array}$$

(14) $$\begin{array}{c} P_{6}^{out}=DSConv\left(\frac{w_{1}^{\prime }∙P_{6}^{in}+w_{2}^{\prime }P_{6}^{td}+w_{3}^{\prime }∙Resize\left(P_{5}^{out}\right)}{w_{1}^{\prime }+w_{2}^{\prime }+w_{3}^{\prime }+\epsilon }\right)\text{,} \end{array}$$

*P*_6_^*td*^ is the intermediate feature of the top-down path of level P_6_, *P*_6_^out^ is the output feature of the bottom-up path of level P_6_, Resize() denotes the upsampling or downsampling of adjusting features of different resolutions to the same resolution, and DSConv is the depthwise separable convolution.

### 3.5. Head: YOLOX

**Figure 9:**
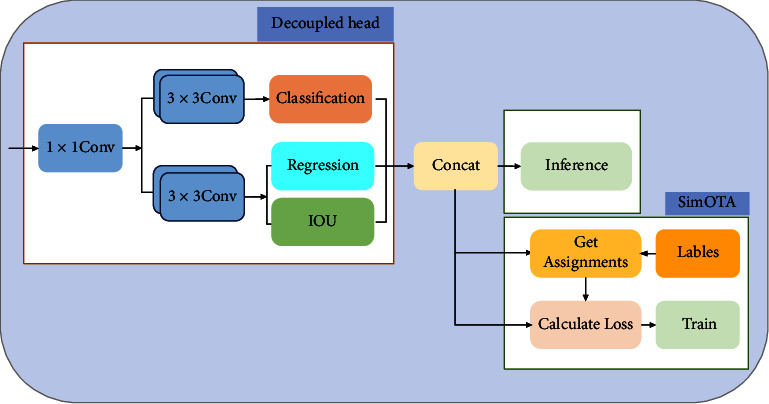
YOLOX head.

YOLOX [2] uses the anchor-free mechanism, decoupling head, and label assignment strategy SimOTA, which simplifies the training and decoding process of the detector while improving the detection precision. Its structure is illustrated in Figure 9.

YOLOX adopts the decoupled head [48] to decouple the classification and regression tasks into two branches, and it adds an IOU branch to the regression branch. The classification branch predicts the object categories, and the regression branch predicts the coordinates of the center point of the bounding box, while the IOU branch predicts the confidence. Its implementation process is as follows: first, the channel dimension of features is reduced through 1 × 1 convolution, after which the features are passed to two parallel branches, each of which has two 3 × 3 convolutional layers. Subsequently, they are passed to the classification, regression, and IOU branches, respectively. Finally, we connect the results of these three branches to obtain the training or inference result. The decoupled head can not only speed up the convergence, but also improve the detection precision of YOLOX.

The process of switching YOLO to an anchor-free manner is as follows: YOLOX reduces the predictions for each location from three to one and makes the detection head directly predict four values, i.e., two offsets in terms of the left-top corner of the grid and the height and width of the predicted box. It assigns each object a positive sample area centered on the object and predefines a scale range, where all samples within this area are positive samples. This alleviates the extreme imbalance of positive and negative samples during training. The anchor-free mechanism improves the precision of object detection and significantly simplifies the detector.

YOLOX proposes a dynamic Top-k label assignment strategy SimOTA, which optimizes the matching method between the prediction (*p*) and ground truth (*g*). The SimOTA implementation process is as follows: first, SimOTA uses the *get assignments* to obtain the ground true label. SimOTA calculates the pairwise matching degree, and the match is represented by cost for each *p*-*g* pair. For example, the cost between *p*_*j*_ and *g*_*i*_ is calculated as follows:

(15) $$\begin{array}{c} c_{ij}=L_{ij}^{cls}+\lambda L_{ij}^{reg}+L_{ij}^{iou}+L_{ij}^{L1}\text{,} \end{array}$$

where *L*_ij_^cls^, *L*_ij_^reg^, *L*_ij_^iou^, and*L*_ij_^L1^ are the classification loss, regression loss, confidence loss, and *L*1 norm loss between the prediction and the ground truth, respectively. *λ* is the balance coefficient. Then, we select the *k* prediction with the smallest cost in the fixed central region as positive samples. Finally, we assign positive values to the grids corresponding to the positive samples and negative values to the remaining grids. SimOTA improves the detection precision and shortens the training period.

## 4. Experimental Analysis and Discussion

### 4.1. Dataset and Evaluation Metrics

#### 4.1.1. Dataset

We use the COCO 2017 dataset [49], which has 118 K images in the training set, 5 K images in the validation set, and 20 K images in the test set. The COCO 2017 dataset [49] has 12 categories and 80 subcategories, including persons, cars, airplanes, trains, boats, dogs, bicycles, motorcycles, buses, traffic lights, and cats. In the training and test sets, there are 66,808 person images, 12,786 car images, 3,083 airplane images, 3,745 train images, 3,146 boat images, 4,562 dog images, 3,401 bicycle images, 3,661 motorcycle images, 4,141 bus images, 4,330 traffic light images, and 4,298 cat images. This annotation includes object regions, category, bounding box (*x*, *y*, width, and height), segmentation information, number of objects, and image_id. We use the validation set for ablation experiments and the test set for comparative experiments.

#### 4.1.2. Evaluation Metrics

AP represents the average precision, AP_S_ represents the AP of small-scale objects with area < 32^2^, AP_M_ represents the AP of medium-scale objects with 32^2^ < area < 96^2^, and AP_L_ represents the AP of large-scale objects with area > 96^2^. Epoch represents the training period. To calculate AP_50_ and AP_75_, the intersection over union (IoU) is used to set the thresholds of the ground truth and prediction boxes. The formula for IoU can be expressed as follows:

(16) $$\begin{array}{c} IOU=\frac{area\left(B_{p}\cap B_{gt}\right)}{area\left(B_{p}\cup B_{gt}\right)}\text{,} \end{array}$$

where *B*_*p*_ is the predicted box, and *B*_gt_ is the ground truth box. AP_50_ and AP_75_ are calculated as fixed values of IOU=50 and 75, respectively. Param (Parameter) is the number of parameters, representing the model complexity, in M. GFLOPs (Floating-point Operations) is the number of floating-point operations, representing the computational complexity, in G. Infer time (Inference time) is the inference time on GPU, in ms. FPS (Frames Per Second) is the number of frames transmitted per second on GPU, representing the inference speed, and FPS = 1000/Infer time.

### 4.2. Implementation Details

We set up a two-stage training strategy. We train the backbone Swin Deformable Transformer in the first stage. First, we pretrain the Swin Deformable Transformer backbone on the ImageNet-1K dataset [35] for initialization, then train it on the COCO training set. We use the AdamW optimizer [50] and soft-NMS [51]. The initial learning rate is set to 3 × 10^−4^, the weight decay parameter is 0.05, the batch size is set to 16, and a 5× schedule is used to train for a total of 60 epochs. The learning rate decays by 0.1 at 40 epochs and 52 epochs. We train the overall network Swin Deformable Transformer-BiPAFPN-YOLOX in the second stage. We, firstly, conduct a 6 epochs warmup on the COCO training set. We use stochastic gradient descent (SGD) for training. The initial learning rate is set to 2 × 10^−3^ and increases to 2 × 10^−2^ by a factor of 10 at 6 epochs. Then, a learning rate of lr × BatchSize/64 (linear scaling) is employed, with a cosine lr schedule. The weight decay is 0.0005, and the SGD momentum is 0.9. The batch size is 128 on 8 V100 GPUs, for a total of 96 epochs. We use the DeiT [34] data augmentation strategy, adopt Mosaic MixUp, and multiscale training [52] to improve the performance of YOLOX and turn off data augmentation in the last 15 epochs [2]. The implementation of our network is based on MMDetection [53].

### 4.3. Ablation Experiment

To study the effect of different components on the performance of our network, we conduct ablation experiments and report the results in Table 1. The AP and AP_S_ of Swin Transformer-PAFPN-YOLOX are 1.0% and 1.8% higher than that of DarkNet53-PAFPN-YOLOX, respectively, indicating that the Swin Transformer extracts the features more effectively than DarkNet53. The AP and AP_S_ of Swin Deformable Transformer-PAFPN-YOLOX are 0.7% and 1.4% higher than that of Swin Transformer-PAFPN-YOLOX, respectively, indicating that our Swin Deformable Transformer performs better than the Swin Transformer. The final Swin Deformable Transformer-BiPAFPN-YOLOX was 0.6% and 0.5% higher than the Swin Deformable Transformer-PAFPN-YOLOX in AP and AP_S_, respectively, indicating that using BiPAFPN as YOLOX' neck outperforms PAFPN. Compared with the original network, our network has fewer parameters, the model complexity is reduced by 30.1%, the computational complexity is reduced by 40.3%, and the inference speed is increased by 10.0%.

To investigate the effect of using Reconstructed Deformable Self-Attention at different stages on the performance of our algorithm, we conduct ablation experiments. The Swin Transformer has a total of four stages, and we use the Reconstructed Deformable Self-Attention in the first block of each stage. On this basis, we use the Reconstructed Deformable Self-Attention in turn in the second block of each stage. The results are shown in Table 2. When the Reconstructed Deformable Self-Attention is used in all blocks of each stage, AP and AP_S_ are increased by 1.3% and 1.9%, respectively, computational complexity and model complexity are reduced by 47.7% and 44.0%, respectively, while the inference speed is increased by 35.2%. This demonstrates that using the Reconstructed Deformable Self-Attention in all blocks of each stage enables our network to achieve the best results.

We propose two schemes: in scheme (A), we use patch grid points instead of random points; in scheme (B), we let the detection head predict the point box offsets of the patch grid point relative to the center point of the bounding box. To verify schemes effectiveness, we conduct ablation experiments, and the results are shown in Table 3. The AP and AP_S_ of scheme A increase by 0.5% and 0.7%, respectively, the AP and AP_S_ of scheme B increase by 0.6% and 0.9%, respectively. When scheme A and scheme B work together, AP and AP_S_ are increased by 0.9% and 1.4%, respectively, and model complexity is reduced by 36.1%, computational complexity is reduced by 29.6%, and inference speed is increased by 32.3%. This shows that our two proposed schemes can significantly improve the detection precision and inference speed of the network and reduce the model and computational complexities.

### 4.4. Comparative Experiment

#### 4.4.1. Quantitative Analysis

To verify whether our network can effectively shorten the training period while maintaining high performance, we conduct comparative experiments and show the results in Table 4. The Swin Deformable Transformer-BiPAFPN-YOLOX has 1.3% and 1.9% higher AP and AP_S_ than the Swin Transformer-BiPAFPN-YOLOX, respectively, while the training epochs are reduced by 55.4%. Simultaneously, the model and computational complexities are reduced by 44.0% and 47.7%, respectively, and the inference speed is increased by 26.0%. This indicates that the Swin Deformable Transformer-BiPAFPN-YOLOX can effectively reduce training time while maintaining high performance.

#### 4.4.2. Qualitative Analysis

**Figure 10:**
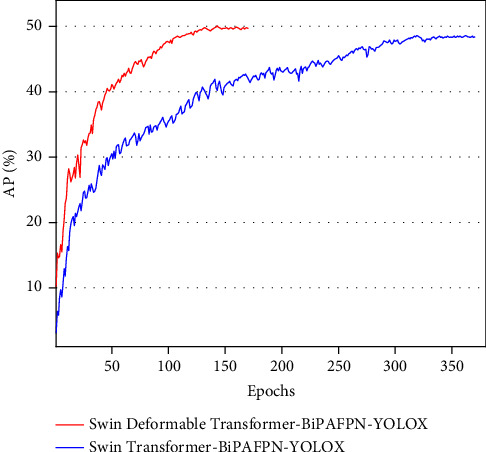
Convergence curves of Swin Deformable Transformer-BiPAFPN-YOLOX and Swin Transformer-BiPAFPN-YOLOX on the COCO 2017 test set.

Figure 10 shows the convergence curves of the Swin Deformable Transformer-BiPAFPN-YOLOX and Swin Transformer-BiPAFPN-YOLOX. The former is represented by the red line, and the latter is represented by the blue line. The Swin Deformable Transformer-BiPAFPN-YOLOX has reached the convergence state around 150 epochs, and its AP is close to 50% at this time; the Swin Transformer-BiPAFPN-YOLOX converges around 350 epochs, and its AP is significantly lower than the former. It also exhibits that the rise of the red line is smoother than that of the blue line. This indicates that our Swin Deformable Transformer-BiPAFPN-YOLOX has faster convergence speed and a more stable training process.

To compare the performance of our network and the original YOLOX [2] network more comprehensively, we expand the parameter scales of our network according to the same scaling rules as YOLOX [2] and obtain four kinds of networks S, M, L, and X that increase in size sequentially. Comparative experiments were carried out on the COCO 2017 test set, and the results are shown in Table 5. The AP and AP_S_ of Swin Deformable Transformer-BiPAFPN-YOLOX are 5.1%, 2.0%, 1.8%, 0.9% and 3.2%, 2.4%, 1.9%, 0.7% higher than the corresponding scale Darknet53-PAFPN-YOLOX, respectively, while the computational complexity is reduced by 39.2%, 31.4%, 7.1%, and 21.4% respectively, and the inference speed is increased by 20.0%, 28.4%, 35.8%, and 22.8%, respectively. This indicates that compared with the original YOLOX [2] network, our network holds higher precision, lower computational complexity, and faster inference speed. The detection precision of small-scale objects is likewise significantly improved.

#### 4.4.3. Quantitative Analysis

To more objectively evaluate the performance of our networks, we compared our four parameter scale networks of S, M, L, and X with other corresponding scale networks. The results are shown in Table 6. At the S level, the AP and inference speed FPS of our network exceed the Swin Transformer v2 by 1.6% (44.7 vs. 43.1) and 44.2% (122.0 vs. 84.6), respectively. At the M level, although the APs of our network is slightly lower than PVTv2 by 0.2% (28.7 vs. 28.9), the AP and inference speed FPS of our network exceed PVTv2 by 2.3% (48.4 vs. 46.1) and 56.4% (104.3 vs. 66.7), respectively, and the computational complexity was reduced by 50.2% (50.6 vs. 101.6). At the L-level, although the AP_L_ of our algorithm is slightly lower than that of the Swin Transformer v2 by 0.5% (66.0 vs. 66.5), the AP, AP_50_, AP_75_, AP_S_, and AP_M_ exceed it by 3.3% (51.8 vs. 48.5), 2.0% (69.6 vs. 67.6), 0.6% (55.4 vs. 54.8), 1.8% (31.7 vs. 29.9), and 1.0% (55.8 vs. 54.8), respectively, and the computational complexity is reduced by 11.8% (181.7 vs. 206.1), the inference speed increased by 72.5% (93.0 vs. 53.9), and the result is similar at the X level. At the X level, the APs of our network exceeds the second best ResNet101-vd-dcn by 0.3% (31.9 vs. 31.6%), the AP exceeds it by 1.8% (52.1 vs. 50.3), and the inference speed increases by 19.7% (71.0 vs. 59.3), while the computational complexity is reduced by 15.6% (225.4 vs. 267.1). The above experiments demonstrate that our network outperforms other state-of-the-art 2D object detection networks in terms of detection precision, inference speed, and computational complexity; it performs well even for small objects that are difficult to detect.

#### 4.4.4. Qualitative Analysis

**Figure 11:**
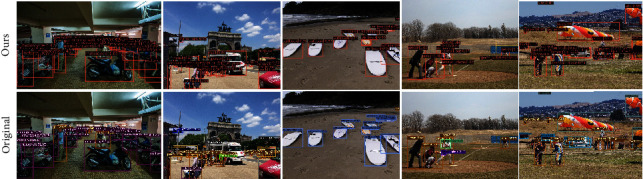
Qualitative experimental results of Swin Deformable Transformer-BiPAFPN-YOLOX (expressed as ours) and Darknet53-PAFPN-YOLOX (expressed as original) on multi-scale objects.

**Figure 12:**
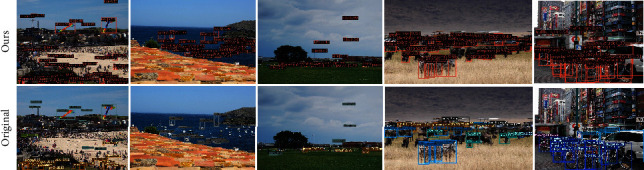
Qualitative experimental results of Swin Deformable Transformer-BiPAFPN-YOLOX (expressed as ours) and Darknet53-PAFPN-YOLOX (expressed as original) on small-scale objects.

**Figure 13:**
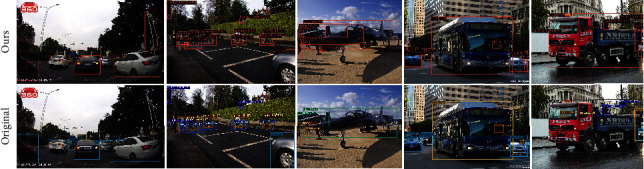
Qualitative experimental results of Swin Deformable Transformer-BiPAFPN-YOLOX (expressed as ours) and Darknet53-PAFPN-YOLOX (expressed as original) on large-scale objects.

To qualitatively evaluate the performance of the Swin Deformable Transformer-BiPAFPN-YOLOX (expressed as Ours) and Darknet53-PAFPN-YOLOX (expressed as Original), we visualize the experimental results of the two networks. Figures 11–13 show the visualization results of the two networks on multi-scale, small-scale, and large-scale objects, respectively. Figures 11 and 12 show that Ours exhibits higher precision on multi-scale and small-scale objects, and yields better results even when the objects are extremely crowded and occluded by each other.  Figure 13 shows that in original, there are some missed detection objects (False Negative) and falsely detected objects (False Positive) when the objects occlude from each other. Hence, our network boasts higher precision on large-scale objects, indicating that it is more advanced.

**Figure 14:**
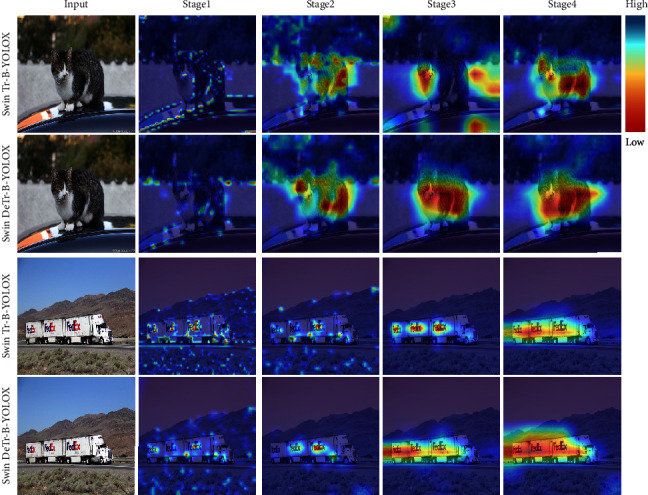
Attention maps of Swin Transformer-BiPAFPN-YOLOX and Swin Deformable Transformer-BiPAFPN-YOLOX on the COCO 2017 test set. Swin Tr denotes Swin Transformer, Swin DeTr denotes Swin Deformable Transformer, and B denotes BiPAFPN.

Class activation mapping (CAM) maps are also referred to as attention maps, and they represent the distribution of the contribution of the prediction result. The important regions on the image are marked as highlighted regions. Brighter regions indicate higher scores, and larger attention weight assigned to them, as well as a more precise prediction result. We adopt Score-CAM [55] to visualize the attention maps of the Swin Transformer-BiPAFPN-YOLOX and Swin Deformable Transformer-BiPAFPN-YOLOX.  Figure 14 shows the attention maps of four stages of the Swin Deformable Transformer-BiPAFPN-YOLOX and Swin Transformer-BiPAFPN-YOLOX. In the first stage, compared with Swin Transformer-BiPAFPN-YOLOX, the attention of Swin Deformable Transformer-YOLOX has a tendency to shift to important regions of the image. In the second stage, compared with Swin Transformer-BiPAFPN-YOLOX, the highlighted regions of Swin Deformable Transformer-BiPAFPN-YOLOX are more concentrated in the object regions of the image, indicating that attention has been shifted to the important object regions. In the third stage, the attention of the first line of Swin Transformer-BiPAFPN-YOLOX is independently distributed at the head and tail of the target, and the attention of the third line of Swin Transformer-BiPAFPN-YOLOX is independently divided into three parts on the object, which causes poor predictions in the fourth stage. In the fourth stage, compared with Swin Transformer-BiPAFPN-YOLOX, the highlighted regions of Swin Deformable Transformer-BiPAFPN-YOLOX completely cover important object regions, and attention has been transferred to the important regions. Larger attention weights assigned to these regions yield more precise prediction results.

## 5. Conclusion

Object detection technology plays a crucial role in people's everyday lives, enterprise production, and modern national defense. This study explores how to apply an attention-based transformer more effectively to the object detection task. We propose an attention mechanism based on important regions, named Reconstructed Deformable Self-Attention, which shifts attention to important object regions, ignores unimportant features, and achieves more efficient global modeling. Based on the Reconstructed Deformable Self-Attention, we propose a novel backbone named the Swin Deformable Transformer, which improves the feature extraction ability and convergence speed of the backbone. Based on the Swin Deformable Transformer backbone, we propose a novel object detection network Swin Deformable Transformer-BiPAFPN-YOLOX. This study further represents the introduction of a Transformer into the object detection network of YOLO series. The experimental results show that compared with the previous state-of-the-art methods on the object detection benchmark of the COCO2017 dataset, our Swin Deformable Transformer-BiPAFPN-YOLOX can significantly boost the detection precision, inference speed, and convergence speed, especially in small object detection. Furthermore, the detected precision increases with the model complexity increases, whereas the inference speed decreases. Thus, high precision and real-time performance cannot be satisfied at the same time. In the future, with the rapid development of computer hardware, we believe that this problem can be efficiently solved. The Swin Deformable Transformer is a multitask backbone. One future research direction is to explore the performance of the Swin Deformable Transformer for segmentation and classification; another is to explore its 3D environment perception performance in multimodal fusion tasks from the perspective of Bird's-eye-view (BEV).

## Figures and Tables

**Table 1:** Results of ablation experiments using different components on the COCO 2017 validation set. Swin DeTr denotes Swin Deformable Transformer.

Methods	AP	AP_50_	AP_75_	AP_S_	AP_M_	AP_L_	Param (M)	GFLOPs	Infer time (ms)	FPS
DarkNet53-PAFPN-YOLOX [2]	47.4	67.3	52.1	27.5	51.5	60.9	63.7	185.3	11.1	90.1
Swin transformer-PAFPN-YOLOX	48.4	67.8	52.6	29.3	52.6	61.8	86.9	221.6	14.5	69.0
Swin DeTr-PAFPN [26]-YOLOX	49.1	68.3	53.0	30.7	54.6	62.9	53.2	173.1	12.4	80.6
Swin DeTr-BiPAFPN-YOLOX	49.7	68.6	53.2	31.2	54.9	63.3	44.5	110.7	10.1	98.7

**Table 2:** Results of ablation experiment using Reconstructed Deformable Self-Attention at different stages on the COCO 2017 validation set.

Stage/reconstructed deformable self-attention								AP (%)	AP_S_	Param (M)	GFLOPs	Infer time (ms)	FPS
Stage 1		Stage 2		Stage 3		Stage 4							
Block 1	Block 2	Block 1	Block 2	Block 1	Block 2	Block 1	Block 2						
✓	✓	✓	✓	✓	✓	✓	✓	49.7	31.2	44.5	110.7	10.1	98.7
		✓		✓		✓		49.6	31.0	51.8	144.3	11.3	88.5
				✓		✓		49.4	30.6	60.3	175.2	11.7	85.5
						✓		48.9	30.3	68.7	193.6	12.9	77.5
Swin transformer-BiPAFPN-YOLOX								48.4	29.3	79.4	211.8	13.7	73.0

**Table 3:** Results of the ablation experiment of the two schemes on the COCO 2017 validation set.

Scheme A	Scheme B	AP (%)	AP_50_	AP_75_	AP_S_	AP_M_	AP_L_	Param (M)	GFLOPs	Infer time (ms)	FPS
		48.8	67.9	52.5	29.8	53.6	62.2	69.6	157.3	13.4	74.6
✓		49.3	68.2	52.8	30.5	54.1	62.7	62.8	148.7	11.7	85.5
	✓	49.4	68.3	53.0	30.7	54.5	62.9	58.5	144.1	11.5	87.0
✓	✓	49.7	68.6	53.2	31.2	54.9	63.3	44.5	110.7	10.1	98.7

**Table 4:** Comparison of training results of Swin DeTr-BiPAFPN-YOLOX, Swin Tr-BiPAFPN-YOLOX and others on the COCO 2017 test set. Swin Tr denotes Swin Transformer, Swin DeTr denotes Swin Deformable Transformer.

Method	Epochs	AP	AP_50_	AP_75_	AP_S_	AP_M_	AP_L_	Param (M)	GFLOPs	Infer time (ms)	FPS
YOLOv4 [3]	300	43.5	65.7	47.3	26.7	46.7	53.3	86.5	223.5	15.6	64.0
YOLOv5 [4]	300	44.5	63.1	—	—	—	—	21.4	51.4	11.1	90.1
DETR [7]	500	42.0	62.4	44.2	20.5	45.8	61.1	41.4	86.9	35.7	28.0
DarkNet53-PAFPN-YOLOX [2]	300	47.4	67.3	52.1	27.5	51.5	60.9	63.7	185.3	11.1	90.1
Swin Tr-BiPAFPN-YOLOX	350	48.4	67.8	52.6	29.3	52.6	61.8	79.4	211.8	13.7	73.0
Swin DeTr-BiPAFPN-YOLOX	156	49.7	68.6	53.2	31.2	54.9	63.3	44.5	110.7	10.1	98.7

**Table 5:** Experimental results of various scales networks on the COCO 2017 test set. Swin DeTr denotes Swin Deformable Transformer.

Models	AP (%)	AP_50_	AP_75_	AP_S_	AP_M_	AP_L_	Param (M)	GFLOPs	Infer time (ms)	FPS
DarkNet53-PAFPN-YOLOX-S [2]	39.6	64.6	47.5	22.7	48.4	54.1	9.0	26.8	9.8	102.0
Swin DeTr-BiPAFPN-YOLOX-S	44.7	67.7	50.3	25.9	50.9	59.6	7.1	16.3	8.2	122.4
DarkNet53-PAFPN-YOLOX-M [2]	46.4	65.4	50.6	26.3	51.0	59.9	25.3	73.8	12.3	81.3
Swin DeTr-BiPAFPN-YOLOX-M	48.4	69.3	53.7	28.7	52.4	61.2	21.2	50.6	9.6	104.3
DarkNet53-PAFPN-YOLOX-L [2]	50.0	68.5	54.5	29.8	54.5	64.4	68.2	195.6	14.5	69.0
Swin DeTr-BiPAFPN-YOLOX-L	51.8	69.6	55.4	31.7	55.8	66.0	63.5	181.7	10.7	93.7
DarkNet53-PAFPN-YOLOX-X [2]	51.2	69.6	55.7	31.2	56.1	66.1	99.1	286.9	17.3	57.8
Swin DeTr-BiPAFPN-YOLOX-X	52.1	70.4	57.8	31.9	56.9	66.7	85.5	225.4	14.1	71.0

**Table 6:** Comparison of our networks with state-of-the-art methods on the COCO 2017 test set. Bold numbers indicate the best results, and blue numbers indicate the second best results. Swin DeTr denotes Swin Deformable Transformer.

Methods	Backbone	Year	FPS	AP (%)	AP_50_	AP_75_	AP_S_	AP_M_	AP_L_	Param (M)	GFLOPs	Infer time (ms)
YOLOv5 [4]	Modified CSPv5	2021	115.0	36.7	62.4	44.1	20.5	45.7	51.9	7.3	17.1	8.7
YOLOX [2]	Modified CSPv5	2021	102.0	39.6	64.6	47.5	22.7	48.4	54.1	9.0	26.8	9.8
YOLOX [23]	Swin transformer v2	2022	84.6	43.1	64.5	48.6	23.7	49.6	57.9	9.3	34.6	11.8
YOLOX [17]	PVTv2	2022	82.7	42.8	63.9	48.4	23.3	49.0	58.1	9.7	36.8	12.1
YOLOX-S	Swin DeTr (ours)	—	122.0	44.7	67.7	50.3	25.9	50.9	59.6	7.1	16.3	8.2

YOLOv5-M [4]	Modified CSPv5	2021	90.1	44.5	63.1	—	—	—	—	21.4	51.4	11.1
YOLOX [2]	Modified CSPv5	2021	81.3	46.4	65.4	50.6	26.3	51.0	59.9	25.3	73.8	12.3
YOLOv4-CSP [4]	Modified CSP	2020	73.0	47.5	66.2	51.7	28.2	51.2	59.8	26.7	94.4	13.7
YOLOX [23]	Swin transformer v2	2022	70.1	46.6	67.1	51.9	28.6	51.3	59.6	28.1	96.2	14.3
YOLOX-M [17]	PVTv2	2022	66.7	46.1	66.9	50.8	28.9	51.6	60.1	29.5	101.6	15.0
YOLOX-M	Swin DeTr (ours)	—	104.3	48.4	69.3	53.7	28.7	52.4	61.2	21.2	50.6	9.6

Reference [54]	Darknet-53	2021	95.2	44.3	64.6	—	—	—	—	63.0	177.3	10.5
YOLOX [2]	Darknet-53	2021	90.1	47.4	67.3	52.1	27.5	51.5	60.9	63.7	185.3	11.1
YOLOv5-L [4]	Modified CSPv5	2021	73.0	48.2	66.9	—	—	—	—	65.1	188.6	13.7
YOLOX-L [2]	Modified CSPv5	2021	69.0	50.0	68.5	54.5	29.8	54.5	64.4	68.2	195.6	14.5
PP-YOLOv2 [47]	ResNet50-vd-dcn	2021	68.9	49.5	68.2	54.4	30.7	52.9	61.2	68.8	197.6	14.5
YOLOX-L [23]	Swin transformer v2	2022	53.9	48.5	67.6	54.8	29.9	54.8	66.5	74.1	206.1	18.6
YOLOX-L [17]	PVTv2	2022	52.7	48.1	67.8	53.9	29.4	53.3	64.6	74.7	208.4	19.0
Def DETR [8]	ResNeXt-101	2021	50.8	49.0	68.5	53.2	29.7	51.7	62.8	76.4	209.8	19.7
YOLOX-L	Swin DeTr (ours)	—	93.0	51.8	69.6	55.4	31.7	55.8	66.0	63.5	181.7	10.8

YOLOv4 [3]	CSPDarknet-53	2020	64.0	43.5	65.7	47.3	26.7	46.7	53.3	86.5	223.5	15.6
YOLOv5-X [4]	Modified CSPv5	2021	62.5	50.4	68.8	—	—	—	—	87.8	239.0	16.0
PP-YOLOv2 [47]	ResNet101-vd-dcn	2021	59.3	50.3	69.0	55.3	31.6	53.9	62.4	90.7	267.1	16.9
YOLOX-X [2]	Modified CSPv5	2021	57.8	51.2	69.6	55.7	31.2	56.1	66.1	99.1	286.9	17.3
YOLOX-X [23]	Swin transformer v2	2022	40.5	50.6	69.1	56.2	31.1	55.7	67.1	110.7	292.5	24.7
YOLOX-X [17]	PVTv2	2022	38.8	50.1	68.9	54.6	30.4	55.9	65.2	113.4	295.7	25.8
YOLOX-X	Swin DeTr (ours)	—	71.0	52.1	70.4	57.8	31.9	56.9	66.7	85.5	225.4	14.1

## Data Availability

The data used to support the findings of this study are available from the corresponding author upon request.
